# Establishment of a Rat Adjuvant Arthritis-Interstitial Lung Disease Model

**DOI:** 10.1155/2016/2970783

**Published:** 2016-01-04

**Authors:** Liu-nan Song, Xiao-dan Kong, Hong-jiang Wang, Li-bin Zhan

**Affiliations:** ^1^Department of Rheumatology, The Second Affiliated Hospital of Dalian Medical University, Dalian 116021, China; ^2^Combination of Chinese Traditional and Western Medicine Lab, Dalian Medical University, Dalian 116044, China

## Abstract

*Introduction*. Development of an animal model of rheumatoid arthritis-interstitial lung disease (RA-ILD) and improved knowledge of the pathogenesis of RA-ILD may facilitate earlier diagnosis and the development of more effective targeted therapies.* Methods*. Adult male Wistar rats were studied in an adjuvant arthritis (AA) model induced by the injection of Freund's complete adjuvant (FCA). Rats were sacrificed on days 7, 14, 21, and 28 after FCA injection. Lung tissue was obtained for histopathological examination and evaluation of Caveolin-1 (Cav-1) and transforming growth factor-*β* (TGF-*β*1) protein expression levels.* Results*. Pulmonary inflammation was evident in lung tissue from day 21 after FCA injection. Inflammation and mild fibrosis were observed in lung tissue on day 28 after FCA injection. Cav-1 protein expression was significantly decreased from day 7 through day 28 and TGF-*β*1 protein expression was significantly increased on day 28 after FCA injection compared to control (*P* < 0.05).* Conclusion*. We established an AA rat model that exhibited the extra-articular complication of RA-ILD. We identified Cav-1 and TGF-*β*1 as protein biomarkers of RA-ILD in this model and propose their signaling pathway as a possible target for therapeutic intervention.

## 1. Introduction

Rheumatoid arthritis (RA) is an autoimmune disease that affects approximately 1% of the population [1]. RA causes synovitis, pain, swelling of the synovium, and important extra-articular complications, including interstitial lung disease (ILD).

In China, RA-ILD occurs in approximately 28% of RA patients and meantime is associated with substantial morbidity and mortality in general population [2]. RA-ILD manifests as progressive fibrosis of the lung parenchyma. Pathogenesis may result from chronic inflammation following initial injury to the alveolar epithelial lining in the presence of a genetic predisposition [3]. ILD is usually detected in patients already diagnosed with RA, but, in some cases, pulmonary disease may precede the onset of articular disease. Median survival after ILD diagnosis is estimated 2.6 years [4].

RA-ILD is an important complication of RA and has a poor prognosis. Early diagnosis and treatment of RA are critical, as proper treatment may slow or even prevent RA-ILD progression [5]. However, there are no guidelines for the management of patients with mild disease, and effective treatment for patients with RA-ILD is lacking. Current practice is to treat patients with clinically significant symptoms or physiologically or radiographically advanced disease. Existing treatment regimens are variable and usually include corticosteroids with or without cytotoxic agents for a duration of six months.

Development of an animal model of RA-ILD and improved knowledge of the pathogenesis of disease may lead to earlier diagnosis and the discovery of more effective targeted therapies. In this study, we established a rat model of RA-ILD based on the existing adjuvant arthritis (AA) rodent model. We investigated the pathological manifestations of RA-ILD by observing inflammation, collagen deposition in lung tissue, and the expression levels of biomarkers of pulmonary disease, including Cav-1, which is protective against lung injury [6], and TGF-*β*1, which can induce pulmonary inflammation [7].

## 2. Materials and Methods

### 2.1. Experimental Animals

Seventy-five male Wistar rats (age: mean 45 days, range: 40–50 days; weight: mean 200 g, range: 180–220 g) were obtained from Dalian Medical University. Rats were housed five per cage with free access to food and water and were allowed to acclimatize to the environment for three days prior to initialization of the experiment. Rats were kept in an air conditioned room at 22°C and 50% humidity and with a controlled 12 h light/dark cycle. The Department of Science and Technology of Liaoning Province approved the animal study and procedures.

### 2.2. Reagents

Freund's complete adjuvant (FCA) was purchased from Sigma-Aldrich, St. Louis, MO, USA; anti-TGF-*β*1 was purchased from Beijing Bioscience; anti-*β*-actin, anti-Cav-1, and secondary antibodies were obtained from Zhongshan Jinqiao.

### 2.3. Experimental Design

Rats were randomly divided into two groups: an experimental group (*n* = 60) and a control group (*n* = 15). Rats in the experimental group were injected with 0.1 mL FCA administered to the right rear paw; control rats were injected with the same volume of PBS. On days 7, 14, 21, and 28 after injection, 15 rats were sacrificed following anesthesia with 10% chloralic hydras (0.3 mL/100 g). Left lung tissue was removed and stored in 10% formalin for histopathological analyses. Right lung tissue was removed and stored at −80°C until analysis by Western blotting.

### 2.4. Model Evaluation

#### 2.4.1. Characterization of AA in Rats

Body weight and paw swelling were measured prior to the induction of arthritis and on every second day after the injection of FCA until the experiment was terminated. Paw swelling was assessed by measuring the thickness of each rat's right rear paw using vernier caliper. The severity of arthritis was assessed according to the following scoring system: 0, normal, with no macroscopic signs of arthritis; 1, mild swelling and/or erythema; 2, moderate erythema and/or swelling of the joints; 3, severe erythema and/or swelling of joints; and 4, complete erythema and swelling of the joints [8].

#### 2.4.2. Histopathology

1 cm × 1 cm pieces of left lung tissue were submerged in 10% formalin for at least 24 h, embedded in paraffin, and cut into five *μ*m sections. Sections were stained with hematoxylin and eosin (H&E) or Masson's trichrome staining protocol to detect collagen fibers.

#### 2.4.3. Western Blotting

Right lung tissue was cut into 5 *μ*m slices at −30°C. Slices were incubated with total protein extraction solution and PMSF for 30 min on ice and centrifuged at 12,000 g for 5 min. Protein in supernatants was denatured in isopycnic loading buffer for 5 min at 100°C.

20 *μ*L sample (2 *μ*L protein) was subjected to electrophoresis on a 12% SDS-PAGE gel (200 v and 50 mA) and transferred to a polyvinylidene difluoride membrane. The membrane was blocked with TBST buffer containing 5% skimmed milk powder for 3 h. Subsequently, the membrane was washed three times in TBST buffer, incubated with rabbit anti-mouse polyclonal anti-Caveolin-1 (Cav-1), anti-TGF-*β*1, or anti-*β*-actin for 24 h at 4°C, washed, and incubated with HRP-conjugated goat anti-rabbit secondary antibody for 2 h at 21°C. After washing, immunoreactive protein bands were visualized using enhanced chemiluminescence and analyzed with the Gel-Pro Analyzer. Expression levels of Cav-1 and TGF-*β*1 of lung tissue were normalized to those of *β*-actin.

### 2.5. Statistical Analysis

SPSS Statistics v.19 was used for statistical analyses. Data are expressed as means ± SD. Comparisons between groups were evaluated using the independent* t*-test. *P* values < 0.05 were considered statistically significant.

## 3. Results

### 3.1. Adjuvant Arthritis

**Figure 1:**
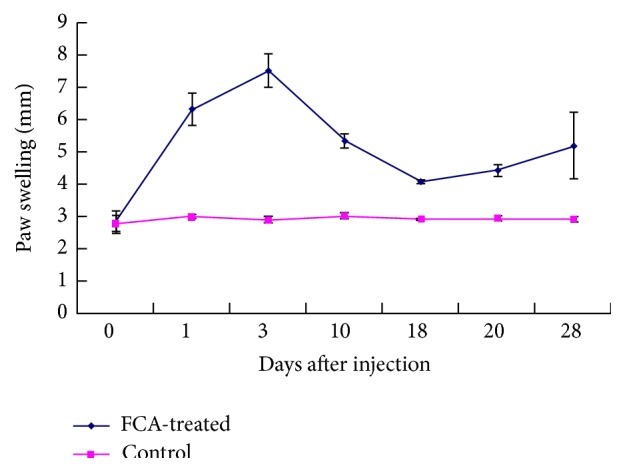
Right rear paw swelling. Mean paw swelling of the FCA-treated rats increased until day three after adjuvant injection compared to control. Subsequent secondary swelling started at day 18.

Signs of FCA-induced AA became apparent 12 h after the injection of adjuvant as the mean right rear paw circumference of the experimental rats increased compared to controls (Figure 1 and Table 1). As AA progressed, the mean circumference of the right rear paw in the experimental rats increased until day 3 and then decreased until day 18. Subsequently, secondary swelling was observed until the experiment was terminated. At each time point, the mean circumference of the right rear paws of the FCA-treated rats was significantly greater than that of the controls (*P* < 0.05) (Figure 1 and Table 1). After 20 days, erythema and paw swelling on the left side could be observed.

Following FCA injection, rats became inactive and depressed, shed their fur, and lost body weight (Figure S1 in Supplementary Material available online at http://dx.doi.org/10.1155/2016/2970783). Mean body weight in the FCA-treated group increased until day 17 and then decreased until the experiment was terminated.

### 3.2. Pulmonary Inflammation and Fibrosis

**Figure 2:**
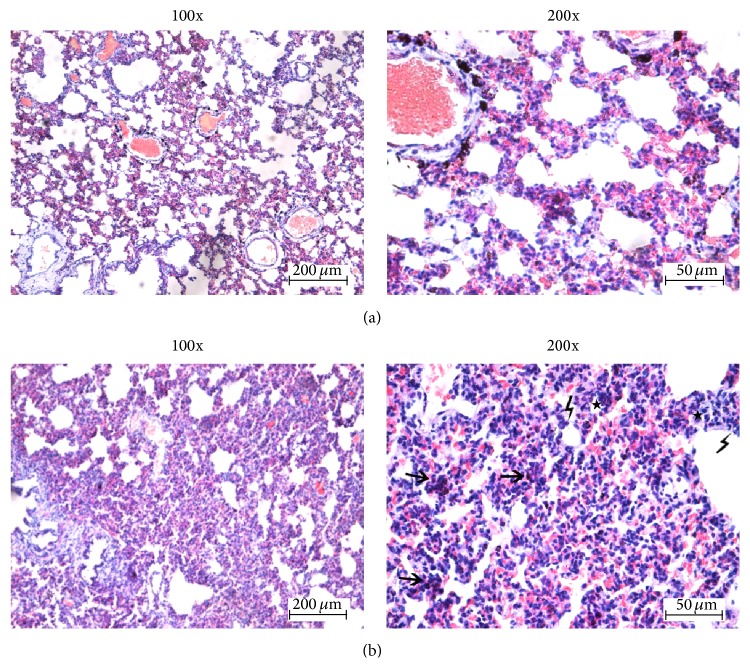
Hematoxylin and eosin stained sections of left lung tissue showing pulmonary involvement 21 days after adjuvant injection.* Right panels* are magnified images of the* left panels*. (a) Control; (b) FCA-treated rat. The alveolar walls are thickened and the structure is generally chaotic. Arrows indicate typical examples of eosinophil and granulocyte infiltration; examples of lymphocyte infiltration are indicated by thunder icons.

**Figure 3:**
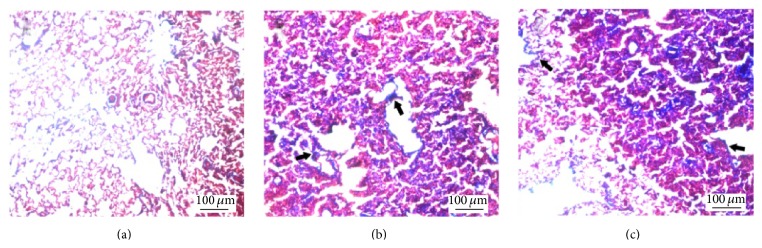
Masson's trichrome stain of left lung tissue showing collagenous deposition (blue stain) 21 days after adjuvant injection. (a) Control: no blue fibers are visible in the interstitial tissue. (b) FCA-treated rat: collagen fibers (arrows) and dense inflammatory infiltration are evident. (c) FCA-treated rat: collagen fibers are close to the pleura (arrows).

**Figure 4:**
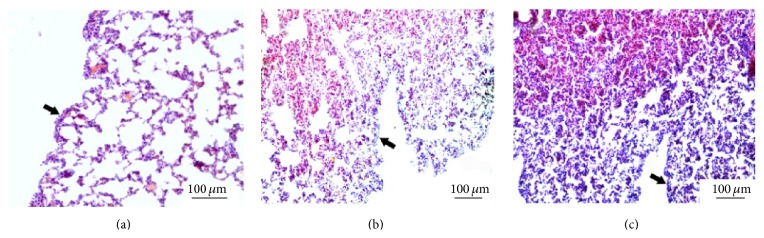
Hematoxylin and eosin staining of left lung tissue showing pleural thickening 21 and 28 days after adjuvant injection. (a) Control. (b) FCA-treated rat 21 days after adjuvant injection: mild pleural thickening (arrows). (c) FCA-treated rat 28 days after adjuvant injection: pleural thickening (arrows).

In FCA-treated rats, H&E staining indicated that the structure of the left lung tissue was chaotic, the alveolar walls were thickened, and there was dense inflammatory infiltration at day 21 after adjuvant injection compared to controls (Figure 2). Deposition of collagen fibers and pleural thickening were seen at days 21 and 28 (Figures 3 and 4).

### 3.3. Cav-1 and TGF-*β*1 Expression

**Figure 5:**
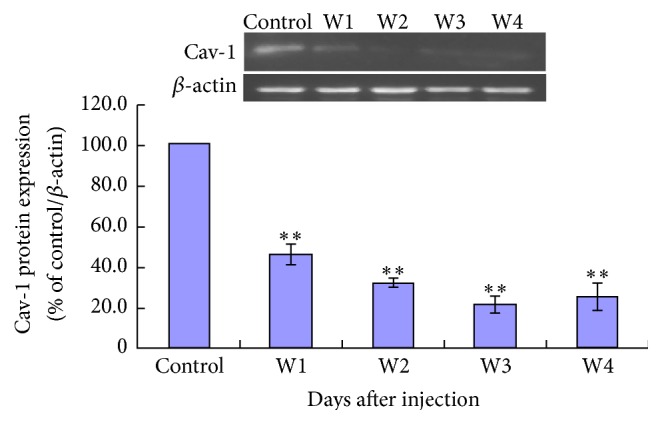
Cav-1 expression of lung tissue detected by Western blotting. On days 7, 14, 21, and 28 after adjuvant injection there was a significant decrease in mean Cav-1 expression level compared to control. ^*∗∗*^ *P* < 0.05, FCA-treated rats versus control.

**Figure 6:**
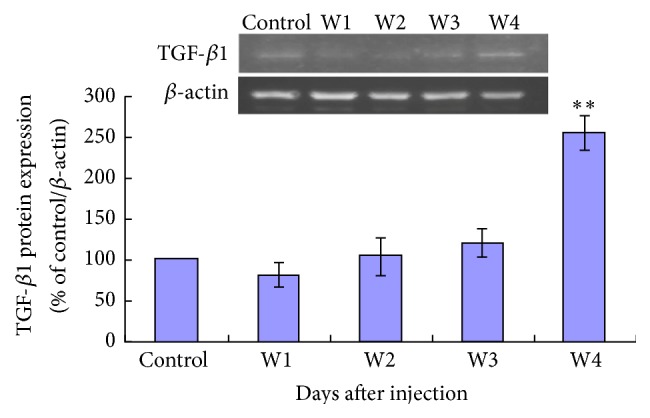
TGF-*β*1 expression of lung tissue detected by Western blotting. On day 28 after adjuvant injection, there was a significant increase in mean TGF-*β*1 expression level compared to control. ^*∗∗*^ *P* < 0.05, FCA-treated rats versus control.

Mean Cav-1 protein expression level of lung tissue was significantly decreased in FCA-treated rats at days 7 (week 1, W1), 14 (week 2, W2), 21 (week 3, W3), and 28 (week 4, W4) after adjuvant injection compared to controls (Figure 5). Mean TGF-*β*1 expression level of lung tissue was significantly increased in FCA-treated rats at day 28 (W4) after adjuvant injection compared to controls (Figure 6).

## 4. Discussion

RA is a chronic systemic autoimmune disease characterized by persistent inflammation of synovial joints and occasional extra-articular complications including pulmonary manifestations such as ILD [9, 10]. The lifetime risk for developing ILD in RA patients is 7.7%, and ILD increases the excess mortality of RA patients by 13% when compared to the general population [4, 11, 12]. Treatment for RA-ILD is often ineffective and is usually restricted to patients with advanced disease. There is little data to guide the treatment of mild disease, as knowledge of the pathophysiology of RA-ILD is limited. Therefore, there is a need for animal models of RA-ILD in which to study the pathological manifestations of the disease and develop effective therapeutic strategies for early intervention.

Bleomycin is widely used to generate animal models of pulmonary fibrosis. However, the mechanism of bleomycin-induced pulmonary fibrosis is different from that of RA-ILD, which limits the use of bleomycin generated animal models in this disease. FCA induces arthritis and pulmonary inflammation in rat [2] and mouse [13] models, probably due to an imbalance between proinflammatory and anti-inflammatory cytokines, which results in inflammation in both joints and lungs. As this inflammatory pattern more closely resembles the clinical characteristics of RA-ILD, we selected an AA rat model to study RA-ILD and investigate the pathological manifestations of the disease.

In our model, injection with FCA resulted in acute paw inflammation until day three and secondary swelling on day 18, indicating that the AA response had been successfully induced. The lung tissues of the FCA-treated rats had a disrupted microscopic structure, and the pulmonary alveoli were infiltrated with eosinophilic granulocytes, lymphocytes, and macrophages. Inflammation was evident in the pleura and collagen fibers were observed close to the pleura. These findings are similar to those seen in RA-ILD patients; therefore, we suggest that our AA rats represent a rodent model of RA-ILD.

ILD is classified into various histopathological subtypes, including usual interstitial pneumonia (UIP) and nonspecific interstitial pneumonia (NSIP) [5, 14]. In patients with RA, ILD subtypes occur at incidences of approximately 56% UIP, 33% NSIP, and 11% organizing pneumonia [15]. We were not able to identify the histopathological subtype of ILD in our AA-ILD rats. More studies are required to describe the complete histopathology of RA-ILD in our model.

We investigated the expression levels of two protein biomarkers of pulmonary fibrosis, Cav-1 and TGF-*β*1, in our AA-ILD rats.* Cav-1* encodes the protein Caveolin-1 and plays an important role in the pathological process of pulmonary fibrosis. Knocking down* Cav-1* increases collagen expression in lung and dermal fibroblasts* in vitro* and* in vivo* [16]. TGF-*β*1 is a multipotent cytokine that can stimulate collagen gene activation, cause deposition of collagen in interstitial tissue, and reduce collagen degradation, eventually leading to fibrosis [17, 18]. Previous studies of a rat bleomycin model of pulmonary fibrosis show that TGF-*β*1 expression peaks at day 6 and day 21 [19] and that overexpression of TGF-*β*1 plays an important role in dermal and pulmonary fibrosis in scleroderma patients [20]. In our rat model of RA-ILD, we found decreased Cav-1 expression levels in lung tissue from day 7 through day 28 after FCA injection. The decreased lung tissue Cav-1 levels coincided with the onset of pulmonary inflammation and increased TGF-*β*1 lung tissue expression levels on day 28 after FCA injection.

Our data are in accordance with previous studies implying an interaction between Cav-1 and TGF-*β*1 in pulmonary fibrosis. Kasper et al. [21] found that Cav-1 expression was decreased in cadmium chloride and TGF-*β*1 treated rat lung. Xiao et al. [6] demonstrated that TGF-*β*1 downregulated Cav-1 in human pulmonary fibroblasts and that Cav-1 was able to suppress TGF-*β*1-induced extracellular matrix production in cultured fibroblasts. Possible mechanisms of interaction between Cav-1 and TGF-*β*1 include the association of Cav-1 with TGF-*β*1 receptor I and inhibition of SMAD2 activation [21, 22]. The role of TGF-*β*1 and Cav-1 in the pathophysiology of RA-ILD and the potential of the Cav-1/TGF-*β*1 signaling pathway as a therapeutic target require further investigation. However, all the data shown in the study cannot illustrate what exactly makes the lung epithelial cells damaged and results in the pulmonary fibrosis. What is the best for the next depth study? We will do the NO releases and Ca^2+^ signaling assay to make sure of the mechanism of the pulmonary fibrosis. Meanwhile, using the Cav-1^−/−^ mice model may be much more useful to the analysis.

In conclusion, we established an AA rat model that exhibited the extra-articular complication of ILD. Because the pathophysiological changes of RA-ILD in this animal model resemble that of human disease, this model will be useful for future research. To the best of our knowledge, this is the first published report identifying Cav-1 and TGF-*β*1 as protein biomarkers of RA-ILD in an AA rat model. The altered expression levels of Cav-1 and TGF-*β*1 in our rat model suggest that Cav-1 and TGF-*β*1 have a role in the pathophysiology of RA-ILD. These results indicate that future therapeutic interventions could potentially target the TGF-*β*/Smad signaling pathway.

## Supplementary Material

Body weight. Mean body weight of the FCA-treated rats decreased 16 days after adjuvant injection compared to control.

## Figures and Tables

**Table 1:** Right rear paw swelling (mm) in FCA-treated and control rats (*n* = 15 at each time point).

Day	0	1	3	10	18	20	28
Model	2.83 ± 0.35	6.33 ± 0.49^a^	7.52 ± 0.51^a^	5.35 ± 0.21^a^	4.09 ± 0.05^a^	4.44 ± 0.18^a^	5.20 ± 1.04^a^
Control	2.80 ± 0.25	3.00 ± 0.06	2.90 ± 0.10	3.03 ± 0.10	2.93 ± 0.02	2.96 ± 0.10	2.93 ± 0.08

^a^ *P* < 0.05, FCA-treated rats versus controls.
