# Supplementary figures and images for: Establishment of a Rat Adjuvant Arthritis-Interstitial Lung Disease Model
Source: Biomed Res Int. 2016 Jan 4;2016:2970783. doi: 10.1155/2016/2970783 (PMC4736313; doi:10.1155/2016/2970783)

## Slide 1
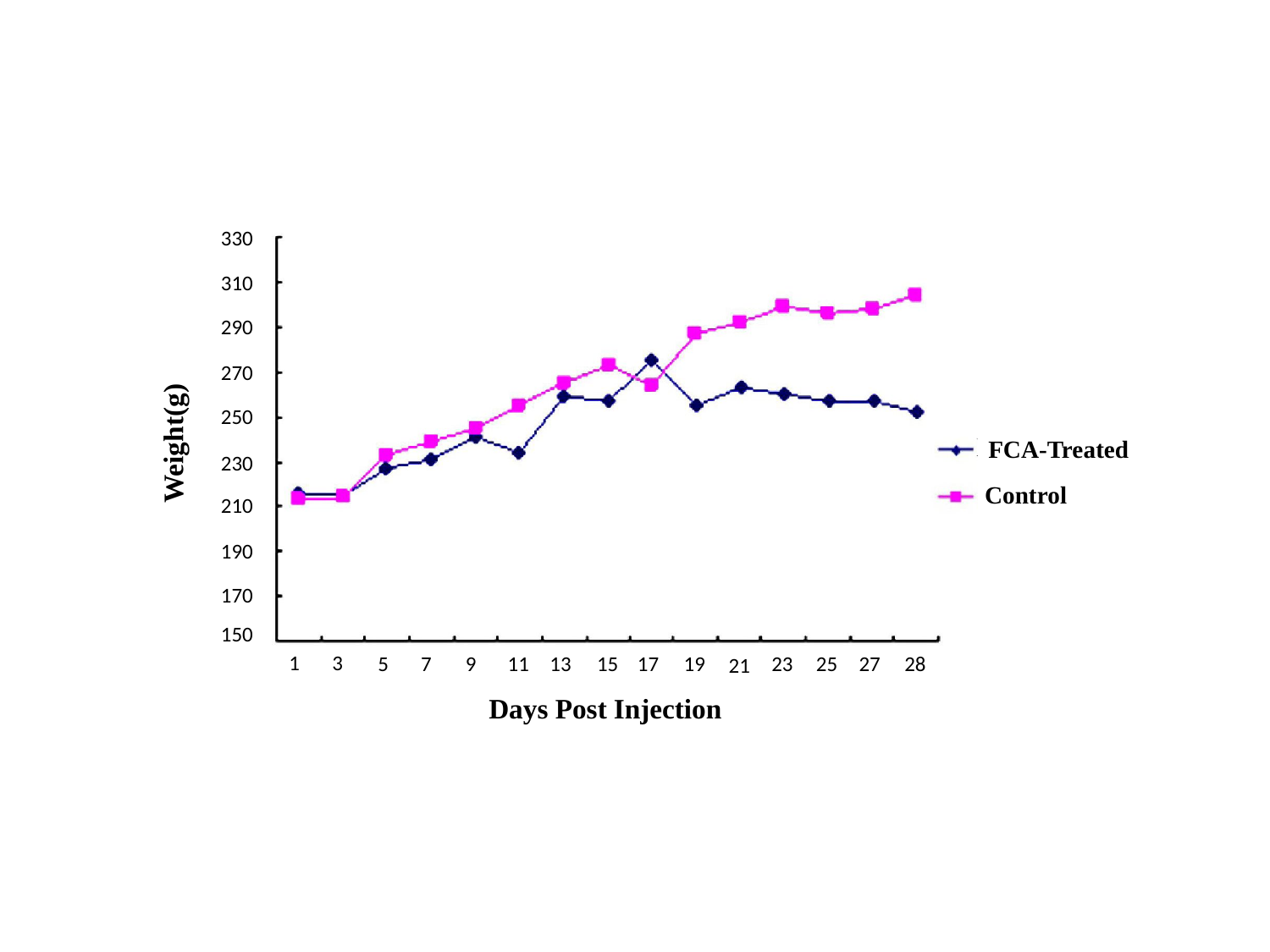

330
310
290
270
Weight(g)
250
FCA-Treated
230
Control
210
190
170
150
1
3
9
5
7
11
13
15
17
19
23
25
27
28
21
Days Post Injection

Supplement: Supplementary file 1 — Body weight. Mean body weight of the FCA-treated rats decreased 16 days after adjuvant injection compared to control. [file 2970783.f1.pptx]
